# Response to Singh and Singh

**DOI:** 10.1038/s41387-022-00195-2

**Published:** 2022-03-30

**Authors:** Philip C. Calder

**Affiliations:** 1 School of Human Development and Health, Faculty of Medicine, University of Southampton, Southampton, UK; 2 NIHR Southampton Biomedical Research Centre, University Hospital Southampton NHS Foundation Trust and University of Southampton, Southampton, UK

**Keywords:** Nutrition, Public health

**Dear Editor,**

I thank Singh and Singh for their interest in my article [1]. However, they misinterpret the message of the article. They summarise that the article “emphasises the need for appropriate nutrition supplementation for the old, frail, obese, diabetic, and generally malnourished, who have been adversely impacted in COVID-19” and they urge caution about micronutrient supplementation in those who are infected. In fact, the summary of my article is that “attention should be focussed on addressing current nutritional inadequacies (frailty, obesity, general undernutrition, micronutrient insufficiency or deficiency) that are widespread in the population in order to better support the immune response …for ensuring the population is better prepared for future pandemics” [1]. With regard to micronutrients, the summary of my article is that “multiple micronutrients play vital roles in supporting all aspects of the immune response and therefore their intake and status need to be considered in the context of susceptibility to SARS-CoV-2 infection and COVID-19 severity” [1]. Thus, the focus of my article is prevention of infectious disease by creating a nutritional environment that supports an appropriate immune response should the individual become infected; the context is not treatment of those already infected. Indeed, my article contains no recommendation to treat those already infected with micronutrients, although it does refer to some studies where vitamin D and zinc have been used as treatments in those with COVID-19. The proposal that several micronutrients are important in supporting the immune system is based upon studies in model systems, including of underlying mechanisms of action, and in humans, as summarised elsewhere [2–4]. Furthermore, this is consistent with the World Health Organisation’s statement in October 2020 that “Micronutrients are critical for a well-functioning immune system, which is of utmost importance during the COVID-19 pandemic. If a population has poor status for key micronutrients, such as vitamin A, zinc or vitamin D, then they may be less well equipped to mount a proper immune response when exposed to viral or bacterial infections than if they had adequate micronutrient status” [5].

The main thrust of the argument of Singh and Singh is that supplementation with micronutrients in those already infected should be approached cautiously. I fully agree with this and with the proposal that the low status of some micronutrients in infected individuals reflects the acute phase response rather than low intakes per se. This is one reason why I avoided making any recommendation for, or against, supplementation in those with infectious disease. Nevertheless, some studies have reported that vitamin D [6–8] and zinc [9, 10] both reduce severity of COVID-19 in hospitalised patients. What is clear is that more needs to be understood about the determinants and meaning of alterations in micronutrient status in those with infections. Thank you to Singh and Singh for drawing attention to this.
